# The Duty of the Physician in Cases of Hopeless Pain

**Published:** 1911-11-04

**Authors:** 


### The Hospital

The Workers' Newspaper of Administrative Medicine and Institutional Life, Administration, National Insurance and Health.

No. 1321, Vol. LI. SATURDAY, NOVEMBER 4TH, 1911. PRICE ONE PENNY.

### THE DUTY OF THE PHYSICIAN IN CASES OF HOPELESS PAIN.

There is no phase of the excessive sentimentality of an ever-increasing number of people in these islands in the present day which is more remarkable than the hurry with which the defective and dependent, the orphan and the innocent destitute, are placed in public institutions, and once there are usually and often speedily forgotten. Yet another phase is that, whereas the absolutely deserving and innocent poor are too often but sparsely provided for, the criminal, the lunatic, the degenerate in character and moral control are well provided for, and are permitted to cost more and more each year to the community. Thus we have the anomaly of people sending their relations into a lunatic asylum where they often fail even to visit them, whilst the ratepayers are called upon by the health authorities to add immeasurably to the expense of these establishments by taking precautions to reduce the death-rate from every cause to a minimum, as if the inmates were the most valuable of lives to the nation. It is time, we think, that some real attempt based upon knowledge and brain-power should be made to remove these contradictions and anomalies. We want a voice to arouse the community to the degeneracy which such anomalies clearly indicate, and in this sense, if for no other, Maeterlinck's brochure on death may be welcomed.

The question as to our duty in cases of hopeless pain is a debatable question of perennial interest, because of constantly occurring examples. When it is a favourite horse or dog we mercifully end its sufferings, and a logical satisfaction compensates in some degree for the painful memory of the ordeal. But when it comes to applying the same principles to human beings, we are immediately conscious of a thousand and one difficulties; our minds are beset by doubts, doubts of prognosis, doubts engendered by religion, doubts stimulated by our views on the sanctity of human life, doubts strengthened by the whole trend of medical training, and the dicta that our business is to save, not to destroy. The feeling is so strong that we disregard even the express wishes of the patient, hardening our hearts against the helpless cry—" Let me die. Let me pass peacefully to the beyond." There is scarcely one of us who has not heard that cry in the course of his career, and has not had to find reasons to himself for the faith that is in him.

Recently the question has been raised and discussed with all the grace, poetical charm, and metaphysical subtlety we associate with the name of Maeterlinck (" Death," by Maurice Maeterlinck, Methuen, 1911), and his final judgment, we find, is against the law, the doctors, and the Church. Death in itself, he maintains, is not terrible; it is the gloom, the misery, the faces of woe that precede it, that have given it the character which we associate with the name. The angel Azrael, coming on silent, soft, grey wings, merciful, compassionate, is not a figure to shrink from. It has been the priests and doctors, according to Napoleon, who have made death grievous. This fear of death is an exotic thing. Essentially it is not native to the Teutonic or Scandinavian mind. It is from the Hellenic, and more particularly the Semitic mind, that we acquire it. The medieval church fostered it; it has been woven into the fabric of our consciousness by the hands of dead theologists. To the Mohammedan, the Buddhist, the Taoist, the Shintoist, death has no terrors; it is but a messenger of a wider, greater world beyond the veil, guiding with courtly care from the finite to the infinite.

We must admit the logic is with Maeterlinck; but for a mere finite being to presume to move the lever leading to the unknown—who is there of us, who, when it came to his own, would willingly curtail one moment of his allotted span! The responsibility is too great. Who can say what case is hopeless? Who of us is able to appreciate the balance of profit between life and death sufficiently accurately to decide " this man must die." Imagination shrinks from it. We can but compromise. We can, by the blessed anodyne of drugs, rob death of its greater agonies. More we dare not do, notwithstanding the fact that logic and commonsense, humanity and charity, appear at times to prompt us to disregard the stereotyped dictates of conventionality and to assuage in a more prompt and thorough manner the agony of suffering for which modern medical science has no cure or permanent alleviation.

